# Oral Treatment of Lactoferrin Nanocapsules Modulates the Immune Response of Mice to a Cryptosporidiosis Infection

**DOI:** 10.1007/s11686-025-01022-1

**Published:** 2025-05-16

**Authors:** Ibrahim Aly, Waleed E. Elawamy, Hanan T. Hamza, Hany M. El-Wahsh, Ahmed EL-Bahiry, Amira Matter, Lamia I. Bakr

**Affiliations:** 1 Parasitology Department, Theodor Bilharz Research Institute, Cairo, Egypt; 2 Department of Pathology (Microbiology Unit), College of Medicine, Jouf University, 72341 Sakaka, Aljouf Saudi Arabia; 3 Department of Parasitology, Faculty of Medicine, Benha University, Benha, 13512 Qaliobyia Egypt; 4 Biology Department, College of Science, Jouf University, 2014 Sakaka, Saudi Arabia; 5 Department of Zoology, Faculty of Science (Girls), Al-Azhar University, Yousef Abbas Str., Nasr City, Cairo, Egypt; 6 Marine Biology Department, Faculty of Marine 555, King Abdulaziz University, Jeddah, Saudi Arabia; 7 Zoology Department, Faculty of Science, Tanta University, Tanta, Egypt; 8 Parasitology Department, Faculty of Medicine, Al-Azhar University, Cairo, Egypt

**Keywords:** Cryptosporidiosis, Drug therapy, Nanazoxid, Lactoferrin nanocapsule, Cytokines, Histopathological lesions

## Abstract

**Purpose:**

The current study was to evaluate the immunomodulatory impact of either lactoferrin (LF), lactoferrin nanocapsule (LF-NC), or lactoferrin conjugation with nanazoxid (NZ). Moreover, identifying drugs can effectively eliminate and successfully treat the infection with *Cryptosporidium*.

**Methods:**

Sixty male Swiss albino mice were divided into six groups, each of 10 mice. G1 served as the control, and G2 was inoculated with 10^4^ oocysts. Mice were orally administered NZ (200 mg/kg b.wt.), LF (150 µg/kg b.wt.), or LF-NC (150 mg/kg b.wt./day) for a period of six days post-infection. The immunomodulatory potential and drug efficacy were assessed by fecal and intestinal oocyst counts, measuring antioxidant activities and cytokine levels in addition. Intestinal tissues were examined histopathologically.

**Results:**

The cytokines TGF-β, INF-γ, and IL-10 were increased during infection and decreased following therapy. Mutually, the quantity of pathological lesions and oocysts in the ileal tissues was significantly diminished with the NZ plus LF-NC treatment. In addition, a considerable reduction of both fecal and intestinal samples, as well as a notable deterioration in oocyst counts, suggested that treatment with NZ alone or in combination with LF-NC was more effective.

**Conclusion:**

LF or LF-NC was found to exert a potent immunomodulatory effect on infected mice as well as minimize pathological lesions. This therapy approaches a successful therapeutic alternative for the treatment of cryptosporidiosis with few adverse effects.

## Introduction

Cryptosporidium is an important protozoan human and animal pathogen that is both waterborne and foodborne [8]. *Cryptosporidium* is a parasitic infection for the small intestine's epithelium that causes diarrhea and severe illness in immunocompetent and immunocompromised patients [18]. As well, infection with *Cryptosporidium parvumis* common in cattle, buffaloes, goats, sheep, horses, cats, human beings, and other vertebrates [24]. According to recent studies, *Cryptosporidium spp.* causes between 48,000 and 202,000 deaths and 7.6 million infections among young children in low-resource settings [20]. The oocysts of *Cryptosporidium* invade the apical intestinal epithelium, resulting in the development of new oocysts expelled by the host [20]. Newborns are susceptible to infection because their immune systems are immature and can become infected by ingesting small amounts of parasite oocysts. *Cryptosporidium hominis* and *C. parvum* species are infrequently detected in people; person-to-person transmission is most frequently identified in humans [16].

Until now, there has been no effective vaccine to prevent the disease, and treatment options are limited. Over 200 compounds have been assessed in humans and animals for their anti-cryptosporidial acts; no vaccine or even appropriate drug is yet to be present [21]. Nitazoxanide (NZ)is the only Food and Drug Administration-approved treatment for cryptosporidiosis [10]. NZ prevent parasitic anaerobic metabolism, which is crucial for energy production, which relieves clearance of parasitic infection [26].

The identification of natural compounds with antiparasitic activity has been a pivotal aim of alternative therapies against several parasites and other diseases [29–31]. Lactoferrin (LF) is considered an attractive natural compound for its protective properties against a wide range of microbes, and it was found to inhibit the growth of cancerous tumors [27, 32]. LF inhibits the growth of protozoan parasites, such as *Toxoplasma gondii*, *Plasmodium falciparum*, and *Trypanosoma cruzi* [32]. Nanoparticles (NPs) attract more attention in applications for drug delivery and treatment [6]. Nanoparticles were industrialized into smart systems for controlled drug delivery and enhanced bioavailability, enhancing the pharmacokinetic profile of encapsulated therapeutic agents [13, 33]. The present study was conducted to evaluate the immunomodulatory effect of LF or LF-NC or their combination with NZ drugs against cryptosporidiosis in an immunosuppressed mouse model.

## Material and Methods

### Chemicals and Drugs

Nanazoxid was purchased from Medizen Pharmaceutical Industries for Utopia Pharmaceuticals; it was given at 200 mg/kg/day successively for seven successive days. Dexamethasone was purchased from Dexazone, Al Kahira pharmaceutical and chemical industry company. Carboxymethyl sephadex-C50 chromatography was purchased from FPLC (Bio-RAD, USA). Cytokines, including IL-10, INF-γ, and TGF-β ELISA kits, were purchased from QIAGEN Company. Biochemical kits were purchased from Bio-diagnostics Company (Giza, Egypt).

### Collection of *C. parvum* oocysts

Patients attending outpatient clinics at Theodor Bilharz Research Institute (TBRI) Hospital. The accepted experimental group to collect the oocysts of *C. parvum,* they were with diarrhea or gastrointestinal problems. Fresh fecal samples were exposed to a direct smear examination, and modified Ziehl–Neelsen stain [1, 34]. Fecal specimens in three vials were received by the parasitology laboratory at TBRI. The vial contains 2.5% potassium dichromate, 10% formalin, or polyvinyl alcohol (PVA). Specimens submitted in PVA were processed and examined by modified Ziehl–Neelsen acid-fast stain.

### Oocyst’s Preparation and Counting

The samples were examined and then stained with modified Ziehl–Neelsen acid fast stain (MZN), according to Garcia [15]. Briefly, stool samples were centrifuged at 500×*g* for 5 min, and the supernatant fluid was discarded. The sediment was washed twice in 1 ml of phosphate buffered saline (PBS) with centrifugation at 13,000×*g* for 2 min. Fecal debris was eliminated form samples after repeated washing and centrifugation.

Fifty µl of prepared cryptosporidium sample was counted under microscope. All mice groups were orally infected with *C. parvum* oocysts by means of esophageal tubes, excluding the control group, obtained from TBRI under the high-power field (HPF), and stained by Kinyoun’s acid-fast stain. Each animal was infected with about 10^4^ oocysts [12, 14].

### Induction of Immunosuppression in Mice

The immune suppression in experimental rats was achieved by treatment with oral dexamethasone, a synthetic corticosteroid, by oral-gastric gavage for 14 consecutive days at a dosage of 0.25 mg/g/days prior to inoculation with *Cryptosporidium* oocysts [28].

### Preparation of Lactoferrin

Cow milk colostrum samples were collected within 72 h after delivery (Faculty Dairy Farm of Behshahr, Mazandaran). After collection, the colostrum sample was placed in an ice flask and transported to the laboratory. The colostrum was centrifuged at 10,000 rpm for 20 min at 4 °C to separate the cream. The cream was discarded, while the skim (whey) was acidified to pH 4.6 using 2 N HCl and kept for 30 min at 40 °C. Acidic pH and heat caused the precipitation of casein. The acid whey was centrifuged at 10,000 rpm at 4 °C for 30 min, and the precipitate was discarded. The acid whey was neutralized to pH 6.8 with 2 N NaOH, and the neutralized whey was incubated at 65 °C for 30 min for pasteurization. Then the whey was precipitated using ammonium sulphate in two steps and centrifuged at 10,000 rpm at 4 °C for 30 min. After analyzing the proteins, we discard the sediment from the first stage. The second-stage sediment was filtered in phosphate-buffered saline (20 mM). Other proteins like β-lactoglobulin, α-lactalbumin, lactoperoxidase, and LF remain in the precipitation. LF was purified using carboxymethyl sephadex-C50 chromatography. Phosphate buffer (200 mM, pH 7.7) and a linear gradient of NaCl from 0.0 to 0.5 M were used, and LF was exited from 0.4 to 0.5 M of the NaCl linear gradient [23, 35].

### Preparation of Lactoferrin Nano-Capsule

The lactoferrin nano (LF-NC) was prepared using a double emulsion/solvent evaporation technique. The lipids were dissolved in dichloromethane, and the aqueous phase was prearranged by dissolving the LF in PBS containing 30% Kolliphor® P407. Sonication solutions at 15% amplitude for 2 min (pulse: 30–5 s) to obtain the first emulsion. The second emulsion was formed by mixing the first emulsion with a α-Tocopherol PEG solution, sonicating at 20% amplitude for 5 min (pulse: 30–5 s), and evaporating. The subsequent LF-NC was then supplemented with a maturation medium, filtered, and ultracentrifuged for 1 h. LF-NC final concentration was homogenized in Milli-Q® water, measured, and lyophilized. The supernatant was retrieved for further studies [4, 22].

### Experimental Design

Sixty male Swiss Albino mice were randomly assigned to six groups (10 per group) as follows:

G1: uninfected normal group.

G2: Infected with *C. parvum* oocysts (10^4^/mouse).

G3: Infected with *C. parvum* oocysts and treated with NZ (200 mg/kg b.wt./day) seven days post infection and continued for 3 days.

G4: Infected with *C. parvum* oocysts and treated with LF (150 mg/kg b.wt./day) seven days post infection and continued for 3 days.

G5: Infected with *C. parvum* oocysts and treated with LF-NC (150 mg/kg b.wt./day) seven days post infection and continued for 3 days.

G6: Infected with *C. parvum* oocysts and treated with LF-NC and with NZ. Each was injected separately with same doses. Seven days post infection and continued for 3 days.

### Stool Examination

Stool samples were daily collected after infection and examined by staining with modified MZN stain, and *C. parvum* oocysts count was calculated using oil immersion magnification at × 100 magnification [34]. Evaluation of the oocyst shedding of *C. parvum* species in the fecal samples was done by collection, cleaning, and homogenization in PBS. The mean shedding was estimated 12 days post-infection (12th dpi).

### Duodenal Content Examination

Mice were sacrificed on the 12th day to collect the duodenal content, and to homogenize in PBS to estimate and calculate the number of * [C. parvum* oocyst shedding [34].

### Histopathological Examination

The mice were sacrificed at 12 days post-infection [6], the pancreatic tissues were embedded in paraffin wax blocks, and stained at the pathology laboratory in TBRI's. Expert histopathologists assessed the pathological dissimilarities, vascular wall changes, and Langerhans islet changes pattern abnormalities [11, 13].

### Evaluation of Antioxidant and Oxidant Markers

Following intestinal tissue homogenization in PBS and centrifugation at 5000 rpm for 30 min, the supernatants were used to measure the oxidative stress biomarker. Malondialdehyde (MDA), and glutathione (GSH) were measured [17].

## Cytokine measurements

The cytokines were determined using ELISA kits for IL-10, INF-γ, and TGF-β, according to the manufacturer instructions. The blood was collected, and centrifuged to collect the serum, and the supernatant was stored at -80°C.

### Statistical Methods

The findings presented in this study represent the average values obtained from three independent replicates. The data were reported as the mean value ± SDE. The comparison between groups was conducted using a one-way analysis of variance (ANOVA). To estimate the presence of a substantial disparity between means, a Turkey post hoc analysis was performed to compare several groups. In the context of statistical tests, a P value less than 0.05 was deemed to be statistically significant. The data and statistical analysis were conducted using SPSS version 25.

## Results

### Fecal *Cryptosporidium* Oocyst Sheddings

The present study evaluated the impact of LF and LF-NC on the shedding of *C. parvum oocysts* in infected mice in comparison with the reference drug NZ. Table 1 shows the number of *C. parvum oocyst* sheddings and the reduction percentages (R%) in the different studied groups. The mean *C. parvum oocyst* shedding was 247,000 ± 1921in the untreated infected group. Mice that were treated with LF had a non-significant reduction in *C. parvum* to 201,000 ± 1306(9.9%).Mice that were treated with LF-NC had the *C. parvum* oocyst shedding significantly reduced to 197,000 ± 1011 (21.2%) (p < 0.001) compared to the infected mice that were treated with NZ, where the *C. parvum* oocyst shedding was significantly reduced to 54,000 ± 989 (79.1%) (p < 0.001).A significant reduction in *C. parvum* oocyst shedding was observed in a group of mice treated with LC-NC loaded with NZ (82.6%) (p < 0.001).

**Table 1:** The total count of fecal and duodenal *C. parvum oocysts* in the different studied group

Groups	No. of Cryptosporidium oocysts	
	Fecal	Duodenal
Infected untreated	247,000 ± 1921	344,000 ± 3217
Infected/NZ	54,000 ± 989	90,000 ± 1322
Infected /LF	201,000 ± 1306	259,000 ± 1989
Infected /LF-NC	197,000 ± 1011	202,000 ± 1431
Infected /LF-NC/NZ	43,000 ± 762	72,000 ± 1081

Data was expressed as mean ± S.D.

*NZ* Nanazoxid, *LF* Lactoferrin, *LF-NC* Lactoferrin nanocapsule

Reduction percentage (R%). Number of oocysts in infected untreated/number of oocysts in treated group

### Duodenal Content Examination

**Fig. 1:**
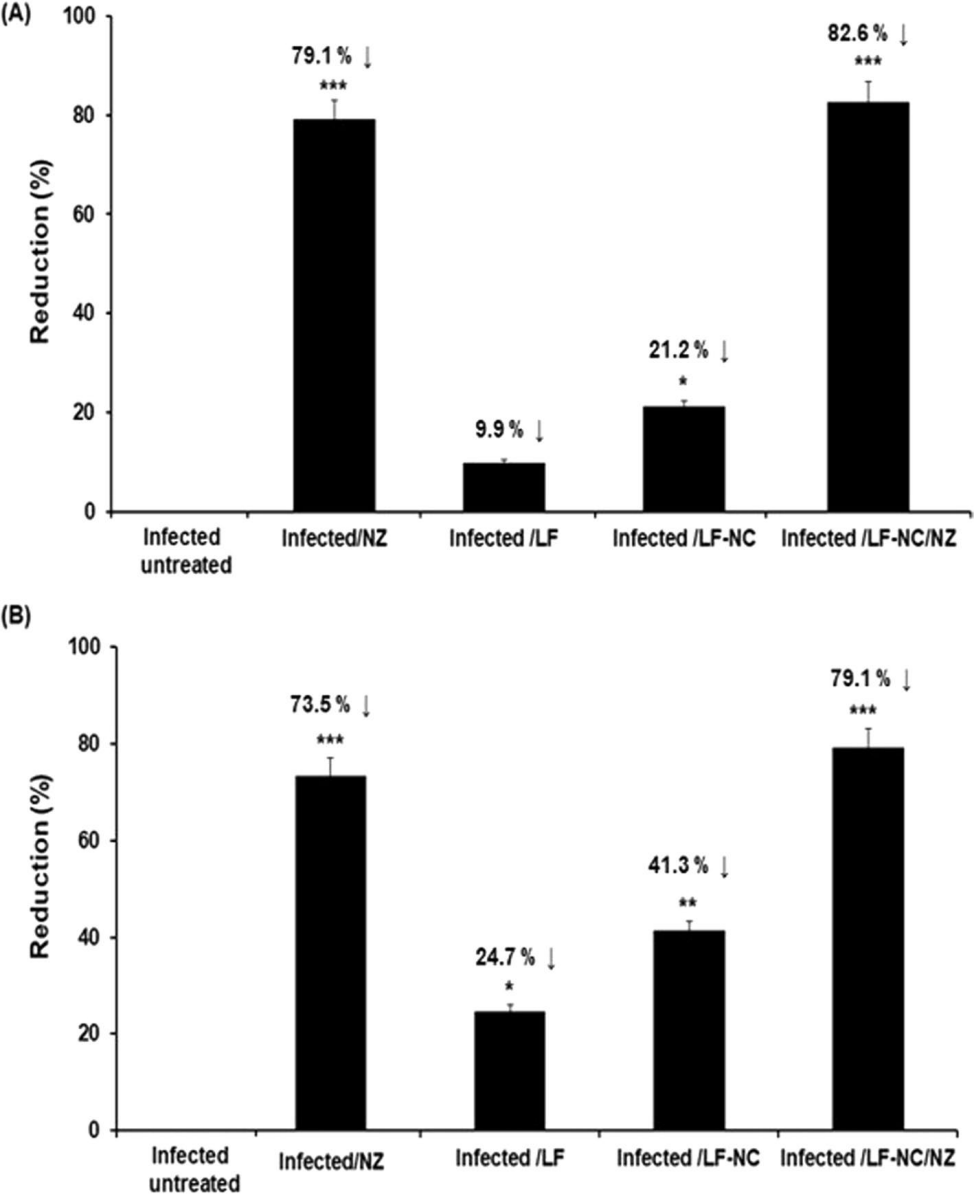
The mean number reduction of *C. parvum oocysts* of the collected intestinal contents mice groups using MZN stain. **A** % reduction in fecal count and **B** % reduction in Duodenal content

The mean number of *C. parvum oocysts* from the collected intestinal contents in each group of mice was calculated using MZN stain. The oocysts were seen as pink, bright-rose ovoid, and round bodies with varying intensity grades against a bluish background. The number of intestinal *C. parvum oocysts* shedding and the R% in different studied groups are illustrated in Fig. 1. The mean *C. parvum oocysts* shedding was 344,000 ± 3217 in the infected, untreated group. Mice that were treated with LF showed a non-significant reduction in *C. parvum oocysts* shedding (259,000 ± 1989,24.7%). The mice that were treated with LC-NC showed a significant reduction in *C. parvum oocysts* to 2202,000 ± 1431 (41.3%) (p < 0.01) compared to treatment with NZ. The *C. parvum oocyst s*hedding was significantly reduced to (91,000 ± 1322, 73.5% (p < 0.001). A significant reduction in *C. parvum oocyst s*hedding was observed in a group of mice treated with LC-NC loaded with NZ (79.1%) (p < 0.001).

### Cytokine Measurements

The level of IL-10 in the infectednontreated group was 49.8 ± 4.5 pg/ml compared to its level in the normal group 4.35 ± 0.012 pg/ml. The infected mice were treated with NZ, LF, or LC-NC and showed different means in concentration levels of IL-10. The mean value of IL-10 was reduced to 12.2 ± 0.57 pg/ml in the group of infected that was treated by NZ. A significant reduction in the IL-10 level was observed in the group that received combined treatment of LF-NC and NZ. The levels of TGF-β in all groups are demonstrated in Table 2. The level of TGF-β was highly significant (p < 0.001) in the infected group (993.7 ± 112 pg/ml) as compared to the normal control (225.3 ± 17pg/ml). TGF-β levels were decreased in all treated groups as compared to the infected group. The maximum reduction in the level of TGF-β was observed in the group of mice that were treated by LF-NC combined with the NZ drug; the level was 331 ± 54 pg/ml, and in the group of infected mice that were treated with the NZ drug, the level of TGF-β was 413.5 ± 36 pg/ml. The mean level of INF-γ in the normal control group was 114.5 ± 12.1 pg/ml, while in the infected group, an increment in the concentration was observed at293 ± 11.5 pg/ml. In general, pre- and post-infection, levels of INF-γ increment were higher when compared to all other groups. The levels of INF-γ in groups of mice that were treated with NZ were 165 ± 16.3 pg/ml and 132.3 ± 14.5pg/ml in the group of mice that were treated with LF-NC combined with the NZ drug (Table 2).

**Table 2:** IL-10, TGF- β, and INF-γ levels were examined in various groups

Groups	IL-10	TGF- β	INF-γ
Control	4.35 ± 0.012	225.3 ± 17	114.5 ± 12.1
Infected	49.8 ± 4.5	993.7 ± 112	293 ± 11.5
Infected/NZ	12.2 ± 0.57	413.5 ± 36	165 ± 16.3
Infected /LF	39.1 ± 5.7	802.9 ± 109	213 ± 22.1
Infected /LF-NC	23.2 ± 3.8	562.3 ± 84	181 ± 28.1
Infected /LF-NC/NZ	9.4 ± 0.72	331 ± 54	132.3 ± 14.5

### Evaluation of Oxidative Stress and Antioxidant Markers

**Fig. 2:**
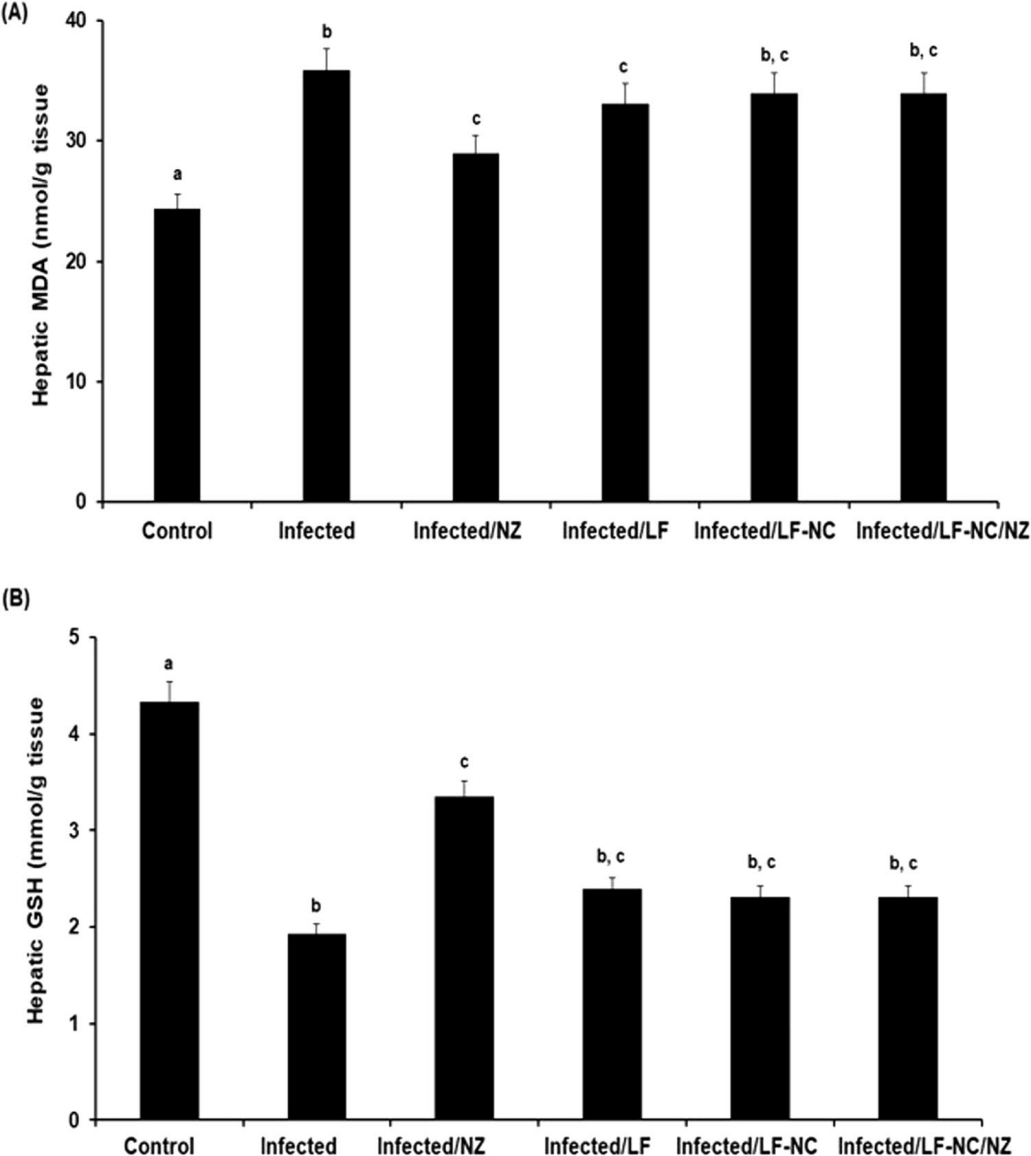
**A** The level of Hepatic Malonaldehyde (MDA) and **B** glutathione (GSH) in experimental groups to measure the oxidative stress and antioxidant markers

The level of MDA in normal control was 21.8 ± 0.678 mmol/g, while it was 45 ± 4.86 mmol/g in the infected control group as depicted in Fig. 2. Moreover, MDA levels decreased in the treated groups in comparison to the infected groups. A significant reduction in the MDA level was reported in the group that was treated with NZ and LF-NC. In addition, GSH level in the normal control group was 4.33 ± 0.179 mmol/g, while a significant decrement was shown in the infected group (1.93 ± 0.115 mmol/g; p < 0.001). The most significant level of GSH was observed in the group of mice treated with NZ and in the group treated with LF-NC (p < 0.001).

### Histopathological Examination

**Fig. 3:**
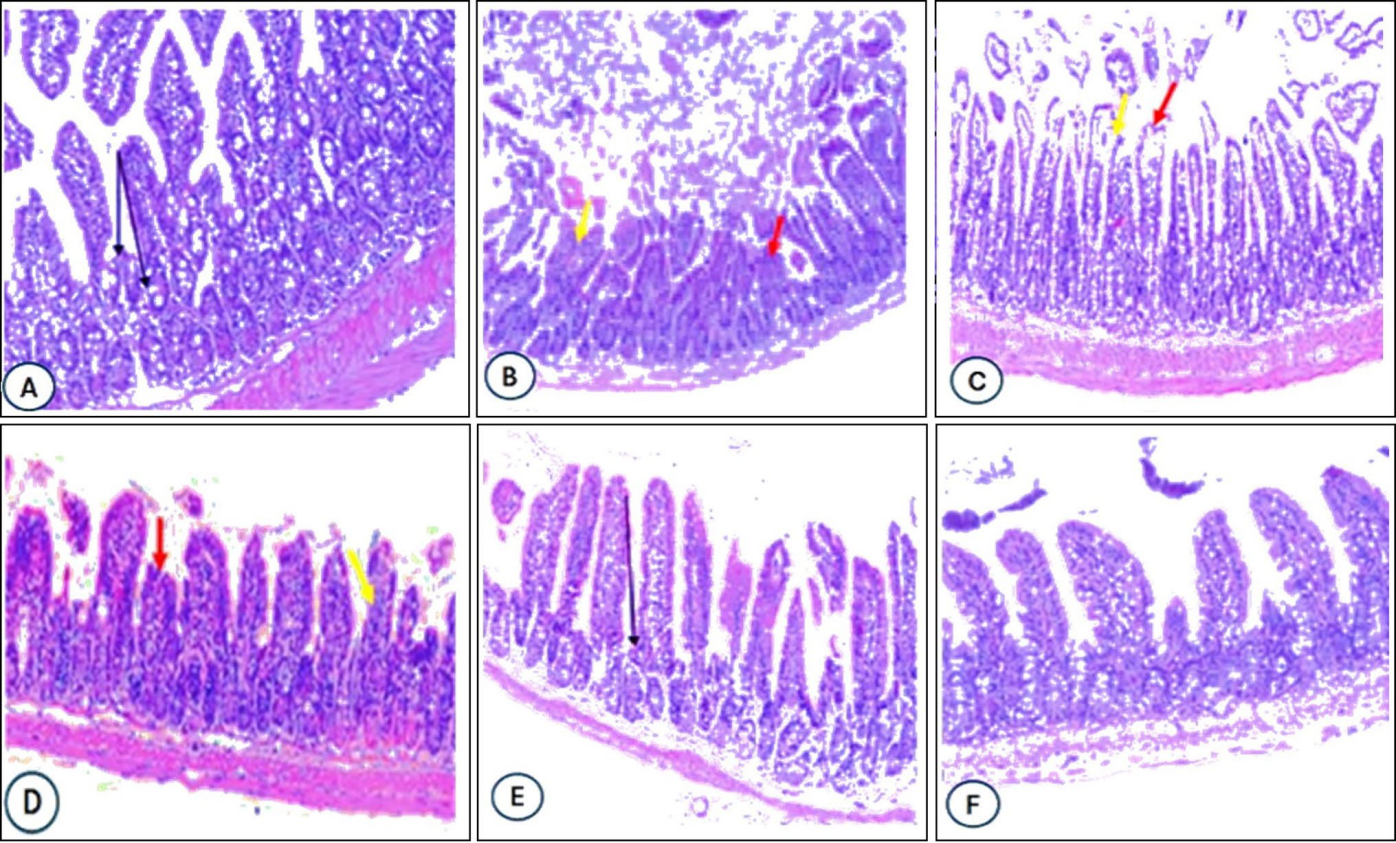
Representative intestinal stained with Hematoxylin and Eosin in various groups. Control uninfected group (**A**), infected control (**B**), infected treated with NZ (**C**), infected treated with LF (**D**), infected treated with LF-NC (**E**), infected treated with LF-NC + NZ (**F**)

Inflammatory changes were detected in * [C. parvum*-infected groups, with a significant dysplastic change were observed in both infected and untreated groups (Fig. 3B). High-grade dysplasia was accompanying to the number of endogenous developmental stages of the parasite, with a higher mean number of oocysts in the infected, and untreated control group (Fig. 3B). Low-grade dysplastic changes were observed in the infected mice and treated with lactoferrin (Fig. 3C), while no dysplasia and no frank carcinoma developed in the groups treated with NZ or LF-NC (Fig. 3D–F).

## Discussion

In the current work, the therapeutic efficacy of nanazoxid (NZ), lactoferrin (LF), LF-NC, and LF-NC/NZ in treating immunocompromised mice experimentally infected with *C. parvum oocysts* was evaluated. Among the indicators used for defining the treatment efficacy of the used drugs was *C. parvum* oocyst shedding and subsequent counts in both stool and intestinal contents and calculating their percent reduction. * [C. parvum* oocyst numbers shed in stools and intestinal contents and their R% among the immunocompromised groups were assessed. It was noticed that * [C. parvum*-infected mice treated with NZ-loaded LF-NC showed the highest percentages of reduction in oocyst shedding (82.6%), followed by a group of mice that were treated with NZ alone (79.1%), in contrast to their equivalent infected untreated group. These findings were consistent with previous data [25]. The highest oocyst shedding was observed in infected and untreated immunosuppressed groups, reaching values of 344,000–3217/g of duodenal contents. This was in parallel with the study conducted by others [5], who disclosed that in severe immunodeficient mice, even when injected with a low parasite load, the parasite excretion increased, reaching mean oocyst numbers of more than 10,000/g feces on the 45th dpi. Correspondingly, another group found that the defeat of the immune system caused by dexamethasone made *C. parvum* organism clearance very problematic, increasing the duration and severity of infection in immunosuppressed mice [36]. However, when NZ was solely administered, it showed 79.1% and 73.5% reduction rates for *C. parvum* oocyst in stool and intestinal contents, respectively, with statistical significance as compared to the control-infected nontreated group. This agreed with earlier researchers, who established the success of NZ in both immunocompetent and immunocompromised groups [2].

Serum levels of IFN-y, TNF-α, and IL-10 were increased in infected and untreated groups. This finding was agreed upon by colleagues, who found that protection against this parasite has been associated with the production of IFN-, TNF-α, and IL-10 [9, 25]. Similarly, IL-10 was an important factor in an efficient anti-* [C. parvum* immune response [19]. The level of hepatic MDA in immunocompromised groups increased in infected groups as compared to normal controls. Hepatic MDA levels were found to be decreased in all treated groups as compared to the infected group. A significant reduction was observed in the group treated with LF-NC + NZ. Our data, in agreement with others, regarding the GSH designation for the liver of immunocompromised groups showed the level of GSH in the normal control group of mice was 4.33 ± 0.179; the infected group showed a decrease to 1.93 ± 0.115 mmol/g. The level of GSH in all treated groups was increased in the group that was treated with LF-NC + NZ [7].

The current study observed that the administration with NZ and LF improves mucosal damage in the infected group. Moreover, inhibit *C. parvum colonization* and multiplication via diminishing anti-cryptosporidial effects. Daily intake of NZ prevents *C. parvum* intestine tissue lesions in immunodeficient mice [3]. In the present study, the liver could also be considered the site of accumulation of the nanoparticles in the mice; in this context, a toxicological study was performed on this organ.

## Conclusion

The current data demonstrated that NZ-loaded LF-NC, due to its non-toxic nature and minor anti-Cyclosporidium action, had the highest significant effectiveness in all subgroups. It is a potential drug that requires continuing efforts to treat cryptosporidiosis in both immunocompetent and immunocompromised humans.

## Data Availability

No datasets were generated or analysed during the current study.

## References

[1] Abdou AG, Harba NM, Afifi AF, Elnaidany NF (2013) Assessment of *Cryptosporidium parvum* infection in immunocompetent and immunocompromised mice and its role in triggering intestinal dysplasia. Int J Infect Dis 17(8):e593–e600 23291034 10.1016/j.ijid.2012.11.023

[2] Abubakar I, Aliyu S, Arumugam C, Usman N, Hunter PJBjocp (2007) Treatment of cryptosporidiosis in immunocompromised individuals: systematic review and meta-analysis. Brit J Clinic Pharmacol 3(4):387–393 10.1111/j.1365-2125.2007.02873.x PMC2203234 17335543

[3] Amadi B, Mwiya M, Sianongo S, Payne L, Watuka A, Katubulushi M, Kelly PJBid (2009) High dose prolonged treatment with nitazoxanide is not effective for cryptosporidiosis in HIV positive Zambian children: a randomised controlled trial. J BMC Infect Dis 9:1–7 10.1186/1471-2334-9-195 PMC2794874 19954529

[4] Balcão VM, Costa CI, Matos CM, Moutinho CG, Amorim M, Pintado ME, Gomes AP, Vila MM, Teixeira JA (2013) Nanoencapsulation of bovine lactoferrin for food and biopharmaceutical applications. Food Hydrocolloids 32(2):425–431

[5] Benamrouz S, Guyot K, Gazzola S, Mouray A, Chassat T, Delaire B, Chabé M, Gosset P, Viscogliosi E, Dei-Cas EJPO (2012) *Cryptosporidium parvum* infection in SCID mice infected with only one oocyst: qPCR assessment of parasite replication in tissues and development of digestive cancer. J PLoS One 7(12):e51232. 10.1371/journal.pone.0051232 PMC3521773 23272093

[6] Boivin GP, Bottomley MA, Grobe NJJotAAfLAs (2016) Responses of male C57BL/6N mice to observing the euthanasia of other mice. J Am Assoc Lab Anim Sci 55(4):406–411 PMC4943610 27423146

[7] Chandramathi S, Suresh K, Shuba S, Mahmood A, Kuppusamy UJP (2010) High levels of oxidative stress in rats infected with Blastocystis hominis. J Parasitol 137(4):605–611 10.1017/S0031182009991351 19961647

[8] Checkley W, White AC, Jaganath D, Arrowood MJ, Chalmers RM, Chen X-M, Fayer R, Griffiths JK, Guerrant RL, Hedstrom LJTLID (2015) A review of the global burden, novel diagnostics, therapeutics, and vaccine targets for cryptosporidium. Lancet Infect Dis 15(1):85–94 10.1016/S1473-3099(14)70772-8 PMC4401121 25278220

[9] Dayao DA, Sheoran A, Carvalho A, Xu H, Beamer G, Widmer G, Tzipori SJIjfp (2020) An immunocompetent rat model of infection with Cryptosporidium hominis and *Cryptosporidium parvum*. Int J Parasitol 50(1):19–22 10.1016/j.ijpara.2019.10.002 31759945

[10] Diptyanusa A, Sari IP (2021) Treatment of human intestinal cryptosporidiosis: a review of published clinical trials. Int J Parasitol Drugs Drug Resist 17:128–138 34562754 10.1016/j.ijpddr.2021.09.001 PMC8473663

[11] El-Sawy SA, Amin YA, El-Naggar SA, Abdelsadik A (2023) *Artemisia annua* L. (Sweet wormwood) leaf extract attenuates high-fat diet-induced testicular dysfunctions and improves spermatogenesis in obese rats. J Ethnopharmacol 313:116528 37127141 10.1016/j.jep.2023.116528

[12] Essendi WMa, Muleke CI, Otachi EO (2024) Concentration and risk assessment of Cryptosporidium infection associated with exposure to the Njoro River, Njoro Sub-County, Nakuru, Kenya. J Basic Appl Zool 85(1):4

[13] Feldman AT, Wolfe D (2014) Tissue processing and hematoxylin and eosin staining. Methods Mol Biol 1180:31–43 10.1007/978-1-4939-1050-2_3 25015141

[14] Gaafar MR (2007) Effect of solar disinfection on viability of intestinal protozoa in drinking water. J Egypt Soc Parasitol 37(1):65–86 17580569

[15] Garcia LS, Brewer TC, Bruckner DA (1987) Fluorescence detection of Cryptosporidium oocysts in human fecal specimens by using monoclonal antibodies. J J Clin Microbiol 25(1):119–121 3539986 10.1128/jcm.25.1.119-121.1987 PMC265837

[16] Gerace E, Lo Presti VDM, Biondo C (2019) Cryptosporidium infection: epidemiology, pathogenesis, and differential diagnosis. Eur J Microbiol Immunol (Bp) 9(4):119–123 31934363 10.1556/1886.2019.00019 PMC6945992

[17] Hassan MH, Awadalla EA, Abd El-Kader AEM, Seifeldin EA, Mahmoud MA, Muddathir ARM, Abdelsadik A (2022) Antitoxic effects of curcumin against obesity-induced multi-organs’ biochemical and histopathological abnormalities in an animal model. Evid Based Complement Alternat Med 2022:9707278 36248416 10.1155/2022/9707278 PMC9560822

[18] Hunter PR, Nichols GJCmr (2002) Epidemiology and clinical features of Cryptosporidium infection in immunocompromised patients. J Clin Microbiol Rev 15(1):145–154 10.1128/CMR.15.1.145-154.2002 PMC118064 11781272

[19] Kaetzel CS (2014) Cooperativity among secretory IgA, the polymeric immunoglobulin receptor, and the gut microbiota promotes host–microbial mutualism. J Immunol Lett 162(2):10–21 10.1016/j.imlet.2014.05.008 PMC4246051 24877874

[20] Khalil IA, Troeger C, Rao PC, Blacker BF, Brown A, Brewer TG, Colombara DV, De Hostos EL, Engmann C, Guerrant RL, Haque R, Houpt ER, Kang G, Korpe PS, Kotloff KL, Lima AAM, Petri WA Jr, Platts-Mills JA, Shoultz DA, Forouzanfar MH, Hay SI, Reiner RC Jr, Mokdad AH (2018) Morbidity, mortality, and long-term consequences associated with diarrhoea from Cryptosporidium infection in children younger than 5 years: a meta-analyses study. Lancet Glob Health 6(7):e758–e768 29903377 10.1016/S2214-109X(18)30283-3 PMC6005120

[21] Khan SM, Witola WHJFic, microbiology i (2023) Past, current, and potential treatments for cryptosporidiosis in humans and farm animals: a comprehensive review. Front Cell Infect Microbiol 13:1115522 10.3389/fcimb.2023.1115522 PMC9902888 36761902

[22] Krzyzowska M, Janicka M, Tomaszewska E, Ranoszek-Soliwoda K, Celichowski G, Grobelny J, Szymanski P (2022) Lactoferrin-conjugated nanoparticles as new antivirals. Pharmaceutics. 10.3390/pharmaceutics14091862 36145610 10.3390/pharmaceutics14091862 PMC9504495

[23] Lu RR, Xu SY, Wang Z, Yang RJ (2007) Isolation of lactoferrin from bovine colostrum by ultrafiltration coupled with strong cation exchange chromatography on a production scale. J Membr Sci 297(1):152–161

[24] Mamedova S, Karanis P (2021) Cryptosporidium spp. infections in livestock and wild animals in Azerbaijan territory. J Water Health 19(4):545–562 34371493 10.2166/wh.2021.050

[25] Mead JR, Arrowood MJ (2013) Treatment of cryptosporidiosis. Cryptosporidium: parasite and disease. Springer, pp 455–486

[26] Putignani L, Menichella DJIpoid (2010) Global distribution, public health and clinical impact of the protozoan pathogen Cryptosporidium. J Interdisc Perspect Infect Dis 2010(1):753512 10.1155/2010/753512 PMC2913630 20706669

[27] Ramírez-Rico G, Drago-Serrano ME, León-Sicairos N, de la Garza MJFiP (2022) Lactoferrin: a nutraceutical with activity against colorectal cancer. Int J Pharmacol 13:855852 10.3389/fphar.2022.855852 PMC8899398 35264972

[28] Rehg JE, Hancock ML, Woodmansee DB (1988) Characterization of a dexamethasone-treated rat model of cryptosporidial infection. J Infect Dis 158(6):1406–1407 3198949 10.1093/infdis/158.6.1406

[29] Salama AF, El-Far AH, Anbar EA, El-Naggar SA, Elshazli RM, Elmetwalli AJMO (2024) Gingerol and/or sorafenib attenuates the DAB-induced HCC and hepatic portal vein dilatation via ATG4/CASP3 and COIIV/COX-2/NF-κB expression. J Med Oncol 41(2): 57 10.1007/s12032-023-02284-3 PMC10791832 38228916

[30] Salama W, El-Naggar S, Harras S, El-Said K (2021) An adjuvant effect of Metformin as an anti-fibrotic agent when administered with the anti-schistosomal Praziquantel in Schistosoma mansoni infected mice. Trop J Pharm Res 20(2):345-350 10.47665/tb.38.2.059 34172712

[31] Salama WM, El-Naggar SAJTJoPR (2021) Cytotoxic effect of Leurius quinquestratus (scorpion) venom in different human cancer cell lines in vitro. Trop Biomed 38(2):205–213 10.47665/tb.38.2.059 34172712

[32] Salatin S, Barar J, Barzegar-Jalali M, Adibkia K, Kiafar F, Jelvehgari MJRips (2017) Development of a nanoprecipitation method for the entrapment of a very water soluble drug into Eudragit RL nanoparticles. Res Pharmaceut Sci 12(1):1–14 10.4103/1735-5362.199041 PMC5333474 28255308

[33] Smith NC, Goulart C, Hayward JA, Kupz A, Miller CM, van Dooren GG (2021) Control of human toxoplasmosis. Int J Parasitol 51(2):95–121 33347832 10.1016/j.ijpara.2020.11.001

[34] Striepen B (2013) Parasitic infections: time to tackle cryptosporidiosis. J Nature 503(7475):189–191 10.1038/503189a 24236315

[35] Superti F (2020) Lactoferrin from bovine milk: a protective companion for life. Nutrients 12(9):2562 32847014 10.3390/nu12092562 PMC7551115

[36] Vanathy K, Parija SC, Mandal J, Hamide A, Krishnamurthy SJTP (2017) Cryptosporidiosis: a mini review. J Trop parasitol 7(2):72–80 10.4103/tp.TP_25_17 PMC5652058 29114483

